# Association between intraoperative blood pressure variability and postoperative complications: a narrative review

**DOI:** 10.3389/fcvm.2025.1737480

**Published:** 2026-01-07

**Authors:** Qian Fu, Yatao Liu, Qijing Liu, Ying Liu, Boxiong Gao, Fang Li, Abdulrahman Khaled Alwesabi

**Affiliations:** 1 The First School of Clinical Medicine, Lanzhou University, Lanzhou, Gansu, China; 2 Department of Anesthesiology and Operation, The First Hospital of Lanzhou University, Lanzhou, Gansu, China

**Keywords:** blood pressure variability, postoperative complications, postoperative delirium, postoperative acute kidney injury, postoperative 30-day mortality rate, stroke

## Abstract

Intraoperative blood pressure variability (IBPV) is an important indicator for perioperative hemodynamic management, and its association with postoperative complications has attracted much attention in recent years. This article reviews the definition, classification, assessment of BPV, and its relationship with postoperative complications, emphasizing the value of real-time BPV monitoring technology in optimizing intraoperative blood pressure management. Existing studies have shown that both elevated and decreased BPV can increase the risk of postoperative complications, but the specific mechanisms and optimal intervention thresholds still need further exploration. In the future, it is necessary to combine artificial intelligence and dynamic monitoring technology to promote the development of individualized anesthesia management strategies.

## 1 Introduction

During the surgical procedure, the patient's blood pressure fluctuations are influenced by multiple factors such as anesthetic drugs, surgical operations, and fluid balance. These fluctuations are not only important indicators for anesthetic management but also closely related to the occurrence of postoperative adverse events. Recent studies have shown that abnormal increases or decreases in IBPV may increase the risk of postoperative complications (1). However, the specific influencing factors of BPV and its pathological mechanism related to complications have not been fully clarified, and there are significant gaps in this field of research. This research gap highlights the importance of real-time monitoring of BPV during surgery, as a deeper understanding of the relationship between BPV and postoperative complications is crucial for improving patient prognosis.

The present review aims to integrate existing evidence to explore the relationship between IBPV and postoperative complications, summarize its clinical significance and current research status, and place particular emphasis on the potential value of real-time monitoring technologies in perioperative management, thereby providing guidance for clinical practice and future research. Previous studies have mostly associated BPV with cognitive impairment and dementia in patients with long-standing hypertension (12, 13), whereas research specifically focusing on IBPV—which reflects short-term blood pressure fluctuations—remains relatively limited. This is partly because most available data have been collected postoperatively, making them insufficient to support real-time intraoperative management. Against this background, real-time monitoring of IBPV becomes especially important, as it allows anesthesiologists to promptly identify and evaluate abnormal blood pressure fluctuations, adjust anesthetic depth, optimize fluid administration, and titrate vasoactive agents. Such dynamic regulation helps maintain blood pressure within individualized target ranges, reduce the incidence of postoperative complications, and improve the safety and effectiveness of surgical procedures.

## 2 Blood pressure variability

### 2.1 Definition

BPV refers to the magnitude or degree of fluctuations in blood pressure over a period of time. It describes the changes in blood pressure values around the mean, rather than a fixed blood pressure value. BPV can be assessed over different time frames: very short-term (sequential measurements), short-term (through ambulatory blood pressure monitoring over 24 h), medium-term (daily home blood pressure monitoring), or long-term (measured at each clinical visit, which can span weeks to years) (Table 1).

### 2.2 Classification (2)

2.2.1 Dispersion[Standard Deviation (SD), Coefficient of Variation (CV), Variability Independent of Mean (VIM)]. SD is the most commonly used metric for measuring BPV, related to the mean blood pressure value and influenced by environmental stressors and circadian variations. Weighted SD is used exclusively for short-term BPV measurement, calculating SD for daytime and nighttime separately and averaging them with weights to eliminate the impact of nocturnal blood pressure decline on SD. CV is used to adjust the relationship between SD and mean blood pressure. CV has stronger prognostic capability than SD because it can identify individuals whose BPV exceeds expected ranges (1). VIM is a transformation of SD that eliminates the influence of mean blood pressure on BPV through nonlinear regression; however, its parameters are specific to each cohort and cannot be compared across populations.

**Table 1:** Common indicators of blood pressure variability.

Indicators	Formulas	Measurement type	Advantages	Limitations	Features
Standard Deviation (SD)	$\mathrm{SD}=\sqrt{\frac{\sum_{i=1}^{n}\;\;{\left(x_{i}-\bar{x}\right)}^{2}}{n-1}}$	Very Short-Term, Short-Term, Medium-Term, Long-Term BPV	Most commonly used;not affected by the measurement order	Sensitive to outliers and nocturnal BP dip	The fundamental metric for overall variability around the mean
Residual Standard Deviation (RSD)	$\mathrm{RSD}=\sqrt{\frac{\sum \;{\left(x_{i}-\bar{x}\right)}^{2}}{n-2}}$	Long-term BPV	Isolates and removes the influence of underlying trends	Assumed model (linear) may not fit complex patterns	Eliminates the influence of long-term trends on variability assessment
Coefficient of Variation (CV)	$\mathrm{CV}=\frac{\mathrm{SD}}{\bar{x}}\times 100\text{\%}$	Short-term BPV	Standardizes variability, allowing comparison across different mean BP levels	May be insufficient if SD and mean are not linearly related	Adjusts for the influence of mean BP on BPV; easy to calculate and interpret
		Medium-term BPV			
		Long-term BPV			
Variability Independent of Mean (VIM)	$\mathrm{VIM}=k\times \frac{\mathrm{SD}}{{\bar{x}}^{m}}$	Long-term BPV	Reduce the influence of the average BP value on BPV	Cohort-specific parameters limit cross-population comparability	Removes the influence of mean BP via non-linear regression analysis
Average Real Variability (ARV)	$\mathrm{ARV}=\frac{1}{n-1}\sum_{i=1}^{n-1}\;|x_{i+1}-x_{i}|$	Short-term BPV	Unaffected by diurnal variation and mean BP value; captures sequential changes	Dependent on continuous measurement data	Simple calculation, good for outcome prediction, more sensitive to sequential variation than SD
		Medium-term BPV			
		Long-term BPV			

2.2.2 Sequence[Average Real Variability (ARV)]. ARV is not influenced by circadian variations or mean blood pressure and is defined as the average of the absolute differences between consecutive blood pressure measurements. It is considered more suitable for measuring 24-hour BPV, as ARV can reveal trends in blood pressure fluctuations during continuous measurements. Compared to SD, it is a more reliable prognostic indicator, as it is more sensitive to individual blood pressure measurement sequences and less sensitive to low sampling frequencies (1).

2.2.3 Instability[Range (Maximum—Minimum BP), Peak Size (Maximum BP), and Trough Value (Mean—Minimum BP)]. The range is the difference between the maximum and minimum values, used to quantify short-term, medium-term, and long-term BPV, and is easily influenced by extreme blood pressure values.

2.2.4 Frequency[Residual Standard Deviation (RSD)]. If there is a linear relationship between time and blood pressure, BPV can be defined as RSD, which is the root mean square error of the difference between predicted and observed blood pressure. This measure is less affected by changes in blood pressure over time, but the assumption of a linear trend does not apply in all situations.

Weighted SD, CV, and ARV were employed to overcome the limitations of conventional SD measurements, providing more accurate evaluations and improved prediction of target organ damage (1). Despite the diversity of assessment methodologies, there remains a notable absence of a unified “gold standard” for BPV quantification, which has contributed to substantial heterogeneity across studies. Consequently, it is imperative to accurately and consistently identify the most reliable metrics while meticulously documenting blood pressure measurements in research protocols. In clinical practice, the selection of appropriate parameters should be guided by individual patient characteristics, with ARV demonstrating particular utility for elderly patients or those with cardiovascular comorbidities.

### 2.3 Measurement methods

Intraoperatively, arterial catheters (radial artery cannulation) are used to achieve real-time arterial blood pressure monitoring. Ambulatory blood pressure monitoring (ABPM) is employed to assess short-term blood pressure fluctuations over a 24-hour period, allowing for unrestricted monitoring of changes at numerous time points during surgery (1). This method can provide high-frequency blood pressure data, typically obtaining a reading every 5 s. Non-invasive cuff measurements can also be used; however, although this method is more convenient, its relatively low measurement frequency cannot fully capture dynamic blood pressure changes, and many brief episodes of high and low blood pressure may be missed with intermittent measurement. The blood pressure monitor records blood pressure data throughout the surgical procedure, which are usually displayed and saved in the form of waveforms. These data can then be utilized for subsequent statistical analysis and evaluation.

### 2.4 Influencing factors

In normal physiological conditions, BPV is primarily influenced by factors such as ventilation, sympathetic nervous system activity, circadian fluctuations in cortisol secretion, increased vascular sensitivity to norepinephrine, diurnal patterns of renin and aldosterone activity, as well as physical activity and sleep habits (4). Under pathological conditions, autonomic dysfunction, persistent activation of the Renin–Angiotensin–Aldosterone System (RAAS), arterial stiffness, and endothelial dysfunction may impair the buffering capacity of the vascular bed against pressure fluctuations, thereby establishing a vicious cycle in which elevated BPV promotes target organ damage and vice versa (31).

The determinants of ARV are multifactorial, involving baseline patient characteristics, perioperative stress, and anesthesia management. Age is one such important factor; ARV tends to increase with aging, suggesting that elderly patients may experience greater hemodynamic variability during anesthetic recovery. In addition, intraoperative blood loss is a key contributor, as greater blood loss predisposes patients to instability during recovery. Factors such as duration of surgery, degree of surgical stimulation, changes in intravascular volume, and certain anesthetic drugs or techniques may also exert direct or indirect influences on ARV (5). Previous studies have indicated that the use of dexmedetomidine, spinal anesthesia or combined regional blocks, and relatively higher doses of opioids in selected high-risk patients can attenuate sympathetic excitation triggered by surgical stimuli, thereby reducing intraoperative blood pressure and heart rate fluctuations and limiting excessive increases in BPV (33–35). Anesthetic depth likewise contributes to IBPV; excessive anesthesia may suppress autonomic responses and generally leads to sympathetic inhibition (6). Evidence further shows that ARV is significantly higher in certain populations, including female patients, individuals with diabetes, smokers, and those with peripheral vascular disease, atrial fibrillation, or a history of transient ischemic attack (TIA) or stroke (7). Among these, patients with autonomic neuropathy (e.g., diabetes or Guillain–Barré syndrome) are particularly vulnerable due to impaired sympathetic–parasympathetic balance and baroreceptor reflex dysfunction. Their diminished ability to adjust to hemodynamic stressors such as anesthetic agents, volume shifts, and positional changes places them at increased risk of blood pressure fluctuations and elevated ARV during the perioperative period (30). These factors, closely linked to patients' physiological and pathological characteristics, contribute to greater uncertainty and heightened risk during anesthetic recovery.

## 3 Relationship between blood pressure variability and postoperative complications

The assessment and quantification of BPV are of great significance for understanding perioperative hemodynamic changes and predicting postoperative outcomes. Existing studies have demonstrated that elevated BPV can independently increase the risk of target organ injury, cardiovascular events, and mortality (3). In non-cardiac surgery, excessively high IBPV often indicates marked hemodynamic instability. Frequent fluctuations in blood pressure, even when not reaching sustained hypotensive or hypertensive levels, may repeatedly expose vital organs—such as the heart, brain, and kidneys—to periods of relative hypoperfusion. Conversely, abnormally low IBPV may reflect impaired autonomic regulatory capacity, whereby patients are unable to adequately adapt to hemodynamic stressors such as changes in intravascular volume or anesthetic depth. For example, IBPV may paradoxically decrease in cases where hypotension persists despite adequate fluid resuscitation and vasopressor administration (1). Therefore, both markedly increased and excessively reduced IBPV may be associated with a higher risk of adverse postoperative events, including acute kidney injury, postoperative delirium, stroke, and 30-day mortality. The evidence supporting these associations will be discussed in detail in the following sections.

### 3.1 Postoperative delirium

In recent years, IBPV has emerged as an important perioperative determinant of postoperative delirium (POD). Unlike absolute hypotension or the duration of low mean arterial pressure (MAP), fluctuations in blood pressure more accurately reflect the integrated response of autonomic regulation and cerebral autoregulatory capacity under the stress of anesthesia and surgery. Consequently, IBPV serves as a more sensitive indicator of transient cerebral perfusion instability. A growing body of evidence across various surgical populations has consistently demonstrated a significant association between IBPV and the development of POD (Table 2).

**Table 2:** Summary of the relationship between blood pressure variability and postoperative delirium.

Author	Year	Meanage	Sample size	Male%	Region	BP index	BP time point	Cognition methods	Cognition time point	Results	Incidence of delirium（%）
Xiao et al. (9)	2024	67	2,164	59.90%	China	SD, ARV	5 min	CAM-ICU	twice a day	Perioperative ARV, especially postoperative high ARV exposure, was associated with POD in the patients receiving cardiac surgery.	15%
Zhang et al. (8)	2023	80	963	24.30%	China	MAPV	30s	medical and nursing records	seven days after surgery	Patients with high intraoperative MAPV are more likely to suffer from POD.	11.90%
Zhang et al. (24)	2016	72	150	40.60%	China	variance	5 min	CAM-ICU, MMSE	three days sfter surgery	The results of the study found that 20% of elderly non-cardiac surgerypatients had POD and 30.4% POCD.	20%
Cai et al. (11)	2023	66	486	63.80%	China	ARV	5 min	CAM, CAM-ICU	four days after surgery	Increased short-term IBPV was an independent risk factor for POD.	14.80%
Peng et al. (25)	2025	79	1,002	42.4%	China	SD, CV	10 min	DSM-5	seven days after surgery	IBPV independently increases POD risk in elderly hip fracture patients	19.8%
Liu et ai. (26)	2025	7/8	522	50%/62%	China	ARV	5 min	PAED	every 10 min until discharged from the PACU	High IBPV and preoperative MRI lesions are independent risk factors for PACU delirium in children with MMD.	37%/25%

In elderly patients undergoing hip fracture surgery, Zhang et al. conducted a retrospective cohort analysis involving 963 cases and found that an intraoperative mean arterial pressure variability (MAPV) exceeding 2.17% markedly increased the risk of POD (OR = 2.379, 95% CI: 1.496–3.771, *P* < 0.001). This association remained robust after propensity score matching (OR = 2.851, *P* < 0.001) (8). The authors proposed that excessive blood pressure fluctuations may induce repeated episodes of cerebral hypoperfusion, leading to microcirculatory dysfunction and metabolic derangements, thereby precipitating delirium more so than hypotension alone.

Comparable findings have been reported in cardiac surgical populations. Shen et al., in a cohort of 2,164 patients undergoing cardiopulmonary bypass, identified a bidirectional relationship between perioperative ARV and POD risk. Both a markedly low intraoperative ARV and an excessively high systolic ARV within 24 h postoperatively were independent predictors of delirium (OR = 1.17, *P* = 0.002) (9). These findings suggest that overly stable hemodynamics may signify impaired cerebrovascular reactivity, whereas excessive postoperative BPV can further disrupt autoregulatory integrity. Both extremes may culminate in cerebral hypoperfusion heterogeneity and neural dysfunction.

The deleterious effects of IBPV are particularly pronounced in patients with compromised cerebrovascular reserve. Liu and He demonstrated that elevated ARV-MAP was an independent risk factor for emergence delirium in pediatric patients with moyamoya disease (OR = 9.17, 95% CI: 3.85–21.82, *P* < 0.001), accompanied by a higher incidence of early postoperative ischemic events (26). This finding underscores that even brief fluctuations in blood pressure can provoke perfusion-metabolism mismatch and neuroinflammatory cascades in individuals with impaired autoregulatory capacity.

Collectively, evidence from cardiac, orthopedic, and neurovascular surgeries converges to highlight IBPV as a critical determinant of POD. The underlying mechanisms are multifactorial, involving disruption of cerebral autoregulation, blood-brain barrier instability, and microcirculatory oxygen imbalance. Excessive fluctuations may trigger ischemia-reperfusion injury and inflammatory activation, whereas excessive suppression of variability may mask inadequate perfusion responsiveness. Both conditions can ultimately impair neuronal integrity and cognition.

From a clinical standpoint, perioperative hemodynamic management should shift focus from merely “maintaining blood pressure levels” to “controlling blood pressure variability.” Efforts should be directed toward minimizing abrupt hemodynamic oscillations while ensuring adequate perfusion pressure. Integrating continuous cerebral oxygen monitoring, individualized fluid optimization, and tailored depth of anesthesia control may collectively mitigate the risk of POD and improve postoperative neurocognitive outcomes.

### 3.2 Postoperative acute kidney injury

IBPV reflects the stability of hemodynamic regulation under surgical and anesthetic stress. Excessive fluctuations in blood pressure can lead to uneven renal microcirculatory perfusion and ischemia–reperfusion injury, thereby serving as a potential trigger for postoperative acute kidney injury (AKI). Unlike sustained hypotension, even when MAP remains within a normal range, frequent oscillations in blood pressure may disrupt renal autoregulation and increase the risk of renal impairment (17, 18).

**Table 3:** Summary of the relationship between blood pressure variability and postoperative acute kidney injury.

Author	Year	Mean age	Sample size	Male%	Region	BP index	BP time point	Evaluation method of AKI	Results	Incidence of acute kidney injury (%)
Park et al. (14)	2020	60	82,659	47.90%	Korea	SD, CV, VIM, ARV	5 min, 2 min, 2 s	The peak serum creatinine within 7 days after operation was 0.3 mg/dL or increased by 50%.	Higher IBPV is associated with a higher risk of AKI after non-cardiac surgery.	4.90%
Folks et al. (15)	2024	57	2,880	58.70%	Korea	SampEn MAP	2 s	KDIGO	The evaluation of very short-term BPV does not improve the ability to identify postoperative AKI in patients undergoing non-cardiac surgery.	7.40%
Packiasabapathy et al. (16)	2020	68	3,687	69.57%	America	CV	15 s	one or both of: 1) increase in serum creatinine level > 2.0, and 2 x greater than baseline, 2) a new requirement for dialysis postoperatively.	BPV computed from Poincare plots and CV were not predictive of mortality and renal failure in cardiac surgical patients.	2.80%
Fishbein et al. (18)	2022	8.6	231	58%	America	ARV, SD	15 s	KDIGO	During cardiac surgery with CPB, elevated BPV is associated with the occurrence of AKI in infants after surgery.	51.50%
Xiao et al.( 19)	2025	1	570	50.70%	China	CV, AUC	5 min	The CSA-AKI was defined as any decrease in estimated creatinine clearance (eCrCl) > 25% from baseline (obtained within 1 week before surgery) to peak value within the first 7 days after surgery	A greater BPV, particularly a MAP change exceeding 30% AUC during CPB, may be a potential risk factor for CSA-AKI in pediatric patients.	36.10%
Jinadasa et al. (20)	2018	68	3,687	69.57%	America	CV	15 s	NA	Among patients undergoing cardiac surgery requiring CPB, we identified a statistically significant association between increased SBPV and renal function failure.	2.80%
Xu et al. (17)	2024	50	437	78%	China	SD, CV, ARV, VIM	1 min	KDIGO	MAP was less than 65 mmHg during liver transplantation.	37.07%
									The time, area, ARV and VIM were significantly correlated with postoperative AKI.	

A growing body of evidence from cardiac surgical populations has demonstrated a significant association between elevated BPV and postoperative renal dysfunction. Jinadasa et al. analyzed 3,687 patients undergoing CPB and reported that each 0.10 increment in the CV-SBP increased the odds of renal failure by approximately 104% (OR = 2.04, 95% CI 1.33–3.14), with the strongest impact observed during the prebypass period; in contrast, variability in MAP did not independently predict renal outcomes (20). Similarly, Packiasabapathy et al. employed Poincaré plots and coefficients of variation to quantify IBPV and found that, while these parameters captured dynamic hemodynamic changes, their predictive accuracy for AKI remained limited, suggesting that BPV should complement rather than replace established perioperative risk models (16). In liver transplantation, the influence of BPV appears even more pronounced. Xu et al. investigated 437 transplant recipients and identified cumulative duration and area of MAP < 65 mmHg, ARV, and VIM as independent predictors of post-transplant AKI (OR = 1.051, 1.008, 1.149, and 2.430, respectively; all *P* < 0.01), whereas greater intraoperative urine output and administration of terlipressin were protective factors (17). Pediatric patients, whose kidneys are developmentally immature and possess limited perfusion reserve, exhibit heightened vulnerability to BP fluctuations. Fishbein et al. reviewed 231 pediatric cardiac surgical cases and found that 51.5% developed postoperative AKI, with both systolic and diastolic ARV independently associated with its occurrence (OR ≈ 1.4, *P* < 0.05), particularly among infants younger than 12 months (18). More recently, Xiao et al. demonstrated in a cohort of children aged 0–7 years that MAP excursions exceeding ±30% of baseline during CPB, quantified as the cumulative area under the curve (±30%AUCm), were significantly correlated with AKI (*P* = 0.038), indicating that excessive pressure oscillations may surpass the threshold of renal autoregulation (19).

Collectively, these studies across diverse surgical contexts consistently highlight that greater IBPV is strongly associated with a higher incidence of postoperative AKI (Table 3). The underlying mechanisms likely involve impaired renal autoregulation, oxidative stress, and microcirculatory injury. Although the quantitative thresholds and metrics used to define BPV (e.g., SD, CV, ARV, VIM) vary across studies, maintaining hemodynamic stability throughout surgery has emerged as a key strategy for renal protection. Future investigations should integrate continuous hemodynamic monitoring with individualized anesthetic management to establish optimal BPV control targets and thereby mitigate the risk of postoperative AKI.

### 3.3 Postoperative 30-day mortality rate

IBPV has emerged as an important hemodynamic marker associated with early postoperative outcomes, particularly short-term mortality. Large-scale studies in both cardiac and non-cardiac surgery have demonstrated that excessive intraoperative fluctuations in blood pressure, independent of MAP levels, significantly increase the risk of death within 30 days postoperatively.

**Table 4:** Summary of the relationship between blood pressure variability and 30-day mortality after surgery.

Author	Year	Mean age	Sample size	Male%	Region	BP index	BP time point	Results	Incidence of 30-day mortality (%)
Jinadasa et al. (20)	2018	68	3,687	69.57%	America	CV	15 s	In patients undergoing cardiac surgery with CPB, we identified a statistically significant association between increased SBPV and 30-day mortality. This is particularly evident in the preoperative period of bypass surgery.	2.70%
Mascha et al. (21)	2015	57	104,401	46.50%	America	ARV-MAP, SD-MAP	1 min	Lower IBPV itself is only slightly associated with postoperative mortality after non-cardiac surgery.	1.30%
Aronson et al. (22)	2010	65	7,504	68%	America	Area under the curve	30 s	IBPV is associated with 30-day postoperative mortality in patients undergoing aortocoronary bypass surgery.	2.10%
Wiórek et al. (23)	2019	48	835	27.70%	Poland	CV	5 min	IBPV may be considered a prognostic factor for the postoperative mortality in non-cardiac surgery, and DBPV seems more accurate in outcome prediction than SBPV	2%
Packiasabapathy et al. (16)	2020	68	3,687	69.57%	America	Poincaré polts, CV	15 s	BPV calculated from Poincare plots and CV did not predict mortality in patients undergoing cardiac surgery.	2.70%

Aronson et al. analyzed 7,504 patients undergoing coronary artery bypass grafting (CABG) and found that greater SBP excursions beyond the optimal range of 105–130 mmHg were strongly predictive of 30-day mortality (OR 1.03 per minute, 95% CI 1.02–1.39, *P* < 0.0001). Among various indices of BPV, the mean duration of systolic excursions outside this range exhibited the highest predictive power, suggesting that both the magnitude and persistence of BP deviations are crucial determinants of adverse outcomes (22). Subsequent studies expanded upon these findings by assessing BPV using more quantitative indices such as the CV. Jinadasa et al. conducted a retrospective study of 3,687 patients undergoing cardiac surgery with cardiopulmonary bypass and demonstrated that an increase of 0.10 in the SBP-CV was associated with a 150% increase in 30-day mortality (OR 2.50, 95% CI 1.60–3.92, *P* < 0.0001). Notably, this effect was driven primarily by the prebypass phase rather than the postbypass period, implying that hemodynamic instability early in surgery exerts disproportionate influence on postoperative survival (20). Similar associations have been observed across broader surgical populations. Benolken et al. analyzed 2,658 adult patients undergoing a variety of major non-cardiac procedures and reported that both preoperative and IBPV independently correlated with adverse outcomes, including 30-day mortality and major complications (adjusted OR 1.42–1.65, *P* < 0.01). Importantly, their study highlighted that BPV exerts prognostic influence regardless of baseline comorbidities or mean pressure levels, reinforcing its potential role as a physiologic marker of cardiovascular resilience (10). In contrast, variability in MAP (CV-MAP) did not independently predict mortality, indicating that SBP dynamics may be more sensitive to physiological stress during anesthesia induction and surgical manipulation. While these results underscore the detrimental effect of large BP oscillations, other investigations have yielded more nuanced conclusions. Packiasabapathy et al. analyzed a similar cohort using nonlinear Poincaré plots and BPV-derived indices (SD1, SD2, CV) and reported that these parameters alone did not significantly improve mortality prediction beyond established risk models such as the Society of Thoracic Surgeons score (C-statistic ≈ 0.7 for both). This suggests that although BPV reflects intraoperative instability, it may serve better as a complementary physiological marker rather than a standalone predictor of outcome (16).

Collectively, the current evidence indicates that elevated IBPV—especially sustained systolic deviations outside individualized optimal ranges—correlates strongly with higher 30-day mortality following cardiac and major non-cardiac surgery (Table 4). The predictive effect appears most pronounced during the early (prebypass) phase, highlighting the importance of early hemodynamic control. However, the integration of BPV metrics into perioperative risk assessment should be contextualized alongside traditional scoring systems and patient comorbidities. Future prospective studies are warranted to determine whether real-time BPV monitoring and targeted anesthetic interventions can effectively reduce perioperative mortality risk.

### 3.4 Postoperative stroke

In recent years, accumulating evidence has underscored the prognostic importance of IBPV in neurosurgical procedures. This issue is particularly salient in revascularization surgery for patients with Moyamoya disease (MMD), in whom impaired cerebrovascular reactivity and limited cerebral blood flow reserve render cerebral perfusion highly susceptible to hemodynamic fluctuations.

**Table 5:** Summary of the relationship between blood pressure variability and postoperative stroke.

Author	Year	Age	Sample size (case)	Male%	Region	BP index	BP time point	Evaluation method of stroke	Stroke time point	Results	Incidence of stroke
Valencia Morales et al. (28)	2023	71	687,581	56%	America	SD, ARV	1 min	ICD-9-CM, postoperative neuroimaging studies (computed tomography and/or magnetic resonance imaging)	Within 72 h postoperatively	IBPV was not associated with increased risk of stroke	0.01%
Li et al. (27)	2020	39	1,497	48%	China	ARV	5 min	CT scan	one day after operation	The high BPV and drastic blood pressure decline are independent risk factors predicting increased risk of postoperative infarction in MMD patients who underwent revascularization surgery.	3.50%
Zhu et al. (29)	2023	7	444	50%	China	ARV	5 min	Evaluated by both the neurologic and CT scan	Within 2 weeks after surgery	The high BPV and drastic decline in blood pressure showed predictive value in postoperative symptom progression.	16%

In a retrospective study involving 1,497 MMD patients, Li et al. demonstrated that marked increases in the ARV of systolic, diastolic, and MAP were significantly associated with a higher risk of early postoperative cerebral infarction, with ORs of approximately 3–4 (27). Furthermore, the maximal declines in MAP and diastolic pressure (max declination) emerged as independent risk factors, suggesting that abrupt BP reductions may precipitate critical drops in cerebral perfusion, subsequently triggering extensive infarction. The investigators further emphasized that BP instability *per se*, rather than absolute hypotension, exerts a more deleterious effect on cerebral hemodynamics. Hence, maintaining hemodynamic stability may be of greater clinical relevance than adherence to rigid upper or lower BP thresholds. A similar pattern has been observed in pediatric populations. Zhu and He et al. analyzed 444 hemispheres from 296 pediatric MMD patients and found that ARV-MAP and maximal MAP decline were both strongly correlated with postoperative TIA progression (OR = 4.731 and 1.271, respectively; *P* < 0.01) (29). These findings suggest that pronounced intraoperative BP fluctuations or abrupt decreases can compromise regional cerebral perfusion, leading to ischemic injury. Given that children possess less mature autoregulatory capacity and are more dependent on hyperperfusion to sustain cerebral oxygen delivery, intraoperative BP instability poses an even greater risk of both functional and structural ischemia. Consequently, maintaining stable hemodynamics during pediatric revascularization is crucial for minimizing postoperative stroke or TIA events. Consistent conclusions were drawn by Valencia Morales et al. in a case-control study, which revealed that patients exhibiting greater intraoperative BP variance not only faced an elevated risk of early postoperative infarction but also tended to develop more extensive lesions. Notably, large territorial infarctions were more often linked to abrupt BP declines, whereas regional infarctions were predominantly associated with sustained oscillations (27). Collectively, these findings indicate that dynamic stability of intraoperative BP may better reflect the equilibrium of cerebral perfusion than its absolute level. For patients with compromised vascular reserve, individualized and continuous BP management strategies should be prioritized over fixed numerical targets.

In summary, excessive IBPV—whether through rapid fluctuations or sudden declines—substantially increases the risk of postoperative cerebral ischemia (Table 5). From a pathophysiological perspective, frequent fluctuations in blood pressure can repeatedly alter shear stress on the vascular wall, imposing mechanical strain and functional injury to the endothelium. This process is believed to promote the formation and destabilization of atherosclerotic plaques, thereby increasing the risk of intravascular thrombosis and ischemic events (32). Maintaining a relatively stable BP trajectory and avoiding transient, sharp deviations, particularly during revascularization or anesthetic transitions, represent key strategies for stroke prevention. Future investigations should integrate cerebral blood flow monitoring with hemodynamic indices to establish individualized BP management models centered on variability control, thereby reducing the incidence of perioperative stroke and improving postoperative neurological outcomes.

## 4 Limitations of current research on intraoperative blood pressure variability and future directions

Although existing studies consistently suggest an association between IBPV and various postoperative adverse events, the reliability of these findings remains limited by substantial heterogeneity across studies. First, significant differences in surgical types, patient characteristics, and anesthetic management strategies lead to fundamental variations in baseline BPV levels and fluctuation patterns. For example, cardiac surgery, liver transplantation, and orthopedic procedures differ markedly in surgical stimulation, fluid shifts, autonomic activation, anesthetic depth, and the use of vasoactive agents; therefore, IBPV values and their associations with outcomes cannot be directly compared across such diverse settings. Second, underlying comorbidities—including diabetes, arterial stiffness, chronic kidney disease, hypertension, and autonomic dysfunction—substantially influence baroreceptor sensitivity and vascular reactivity. These factors may increase BPV and independently elevate postoperative complication risk, creating bidirectional confounding effects. Third, the BPV metrics and monitoring methods used in current studies vary widely, ranging from SD, CV, ARV, and VIM to sampling intervals spanning from seconds to several minutes. These methodological inconsistencies further limit the comparability and generalizability of research results. Finally, as most studies are observational in nature, they can demonstrate associations but cannot establish causality; thus, caution is required when interpreting the relationship between IBPV and organ injury. These limitations collectively influence the rigor of the conclusions drawn in this review and underscore the need for more standardized, mechanistic, and interventional investigations.

Future research should be conducted under more controlled and standardized conditions to address these limitations. First, unified and practical IBPV measurement standards are needed, such as clearly defining blood pressure monitoring frequency, prioritizing more stable BPV metrics, and establishing reference ranges for different patient populations. Such standardization would not only reduce variability across studies but also help clinicians more accurately determine which blood pressure fluctuations may pose risks. Second, further investigation is required to elucidate how IBPV affects critical organs such as the heart, brain, and kidneys. Studies examining microcirculatory perfusion, inflammatory responses, and baroreceptor sensitivity may clarify the mechanisms by which blood pressure fluctuations lead to organ injury, thereby providing a stronger theoretical basis for targeting IBPV in clinical interventions. With the expanding application of artificial intelligence in perioperative care, predictive monitoring has received increasing attention. For instance, algorithms such as the Hypotension Prediction Index (HPI) can issue early warnings before blood pressure declines, allowing anesthesiologists to adjust treatment in advance. Inspired by this concept, future work may involve the development of tools capable of real-time analysis of BPV trends, enabling earlier identification of potential hemodynamic instability and supporting proactive management. Finally, conducting prospective studies in more homogeneous patient populations (e.g., cardiac surgery) or controlled animal experiments will be essential to address a key question: whether stabilizing blood pressure variability alone, after minimizing confounding factors, can truly reduce postoperative complications. If this is confirmed, integrating patients' genetic background, metabolic characteristics, and physiological profiles may facilitate the development of more precise, individualized blood pressure management strategies, ultimately improving perioperative safety and outcomes.

## 5 Conclusion

IBPV is an independent risk factor for postoperative complications, and its effects involve the nervous, renal and cardiovascular systems. Through real-time monitoring and precise intervention, it is expected to improve the prognosis of patients. In the future, multidisciplinary cooperation should be promoted, and basic research, technological innovation and clinical practice should be closely integrated to provide more efficient solutions for perioperative management.
